# Phenotypes of Exacerbations in Chronic Obstructive Pulmonary Disease

**DOI:** 10.3390/jcm14093132

**Published:** 2025-04-30

**Authors:** Lucia-Cristina Nisip Avram, Tamara Mirela Poroșnicu, Patricia Hogea, Emanuela Tudorache, Elena Hogea, Cristian Oancea

**Affiliations:** 1 Doctoral School, “Victor Babes” University of Medicine and Pharmacy Timisoara, Eftimie Murgu Square 2, 300041 Timisoara, Romania; cristina.nisip@umft.ro; 2 Department of Anesthesia and Intensive Care, “Victor Babes” University of Medicine and Pharmacy Timisoara, Eftimie Murgu Square 2, 300041 Timisoara, Romania; mirela.porosnicu@umft.ro; 3 Center of Research and Innovation in Personalized Medicine of Respiratory Disease (CRIPMRD), “Victor Babes” University of Medicine and Pharmacy Timisoara, Eftimie Murgu Square 2, 300041 Timisoara, Romania; oancea@umft.ro; 4 Department of Pulmonology University Clinic, “Victor Babes” University of Medicine and Pharmacy Timisoara, Eftimie Murgu Square 2, 300041 Timisoara, Romania; 5 Department XIV, Discipline of Microbiology, Victor Babes University of Medicine and Pharmacy, 300041 Timisoara, Romania; hogea.elena@umft.ro

**Keywords:** chronic obstructive pulmonary disease (COPD), phenotypes, exacerbations

## Abstract

Chronic obstructive pulmonary disease (COPD) is a chronic respiratory disease with an important public health challenge and a major burden on health-care resources, having a progressive character with constant deterioration of lung function. During the course of the disease, patients experience acute episodes of exacerbation, which are characterized by worsening symptoms, and require additional treatment during these exacerbating episodes. Given the heterogeneity of exacerbations, their phenotyping is of great interest in order to administer the most effective treatment with the aim of reducing mortality and preventing future exacerbation episodes. The lack of specific biomarkers for the diagnosis of acute exacerbations of COPD maintains researchers’ interest in trying to identify such a biomarker. In this review, we explore the different phenotypes of COPD exacerbation, and we also evaluated the ability of various biomarkers to establish the etiology of exacerbations in association with clinical manifestations. Furthermore, we addressed the main therapeutic measures necessary according to each phenotype. Overall, phenotyping exacerbations allows for an individualized approach to these patients, thus avoiding the side effects of some treatments.

## 1. Introduction

Chronic obstructive pulmonary disease (COPD) is a chronic respiratory disease with an important public health challenge and a major burden on health-care resources [1]. Prevalence, morbidity, and mortality vary between different countries and between different population groups. The prevalence of the disease is directly related to the prevalence of smokers, but in some countries it depends on other risk factors such as outdoor, occupational, and household air pollution [2,3,4]. The global prevalence of COPD is estimated to be approximately 10% according to epidemiological studies [5,6]. In terms of mortality, this disease is the 4th cause of death globally, according to World Health Organization (WHO), totaling approximately 5% of the total number of deaths in a year (3 million deaths per year) [7].

## 2. Definitions

The definition of the disease has been adapted over time based on pathophysiological research, aiming to include the most recent advances in the understanding, diagnosis, and management of this complex disease. The current definition is as follows: “COPD is a heterogeneous lung condition characterized by chronic respiratory symptoms (dyspnea, cough, expectoration) due to persistent abnormalities of the airways (bronchitis, bronchiolitis), and/or alveoli (emphysema), that often results in progressive airflow limitation” [5,8].

The course of the disease is progressive, with constant deterioration in lung function. Patients also experience acute exacerbations, which are characterized by worsening symptoms and require additional treatment during these exacerbating episodes. Furthermore, COPD exacerbations (AECOPD) are associated with rapid functional decline, the need for hospitalization, and increased risk for future exacerbations and disease progression, all of which have a negative impact on health status [1,5,9,10].

Over time, COPD exacerbations have been defined in various ways, depending on the symptoms presented by patients, on certain events, and on a combination of these features [11,12,13]. Some of the proposed definitions for AECOPD that have been developed over time are as follows:

- Chest illness that causes loss of time from work or forces the patient to stay indoors or in bed, in association with symptoms presented during an exacerbation—increased sputum volume, sputum color, dyspnea, temperature [14].
- Increases in dyspnea, cough, and sputum production with increased purulence in sputum [15].
- “A sustained worsening of the patient’s condition, from the stable state and beyond normal day-to-day variations, necessitating a change in regular medication in a patient with underlying COPD” [16].
- Exacerbation is associated with refractory dyspnoea (>4 on a 0–10 scale), worse cough and sputum, manifestations of systemic involvement, such as tachypnoea (>24 breaths/min), fever, elevated white cell count (>9000 cells/dL), and CRP (>10 mg/dL), without evidence of infiltrates in the chest radiograph [17].
- “AECOPD are defined clinically as episodes of increasing respiratory symptoms, particularly dyspnea, cough and sputum production, and increased sputum purulence” [18].

To date, there is no specific biomarker or standardized test for the diagnosis of AECOPD. Although attempts have been made to develop a definition based on objective criteria, the most widely accepted and well-known definition is that of the GOLD report [11]. This definition is based on clinical, subjective elements, such as dyspnea, cough, and sputum appearance, as well as on paraclinical elements such as inflammation, defining COPD exacerbation as follows “an AECOPD is defined as an event characterized by increased dyspnea and/or cough and sputum that worsens in <14 days which may be accompanied by tachypnea and/or tachycardia and is often associated with increased local and systemic inflammation caused by infection, pollution, or other insult to the airways” [5].

**Figure 1:**
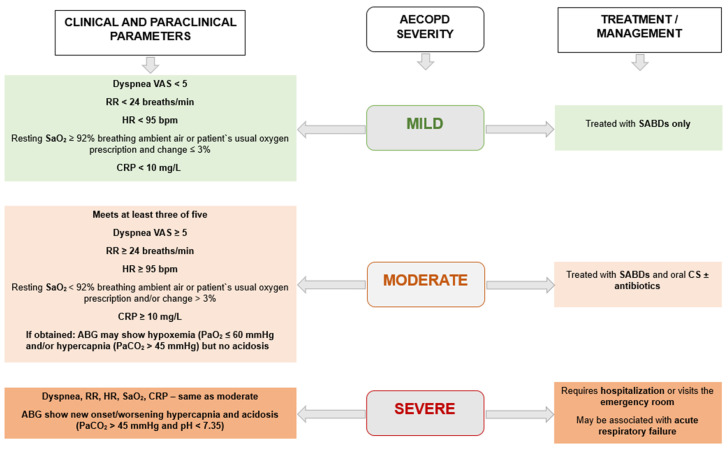
The severity of AECOPD (adjusted according to GOLD 2025 and Celli BR. et al., 2021 [5,19]). Abbreviations: VAS—visual analog dyspnea scale; RR—respiratory rate; HR—heart rate; SaO_2_—oxygen saturation; ABG—arterial blood gases; PaO_2_—arterial pressure of oxygen; SABD—short acting bronchodilators; CS—corticosteroids.

The classification of patients based on the severity of AECOPD is conducted based on clinical and paraclinical parameters, as well as the treatment administered. The Rome classification of exacerbations proposed the use of clinical variables to establish the severity of the exacerbation, these variables being easy to obtain regardless of the facilities of the healthcare facility where the patient presents. Also, where possible, the clinical part will be complemented with paraclinical investigations such as C-reactive protein, blood gas analysis, or antibiogram, as can be seen in Figure 1, these data being useful in establishing the severity of the exacerbation and establishing the etiology and treatment. The classification of COPD exacerbations according to the ROMA proposal correlates with the prognosis and mortality of patients. Of major importance is the differential diagnosis, especially with heart failure, pneumonia, or pulmonary embolism, by corroborating the symptoms with the results of specialized paraclinical investigations [19,20,21]. Figure 1 shows the severity of AECOPD, adjusted according to the ROME proposal, GOLD 2025, and Celli BR. et al., 2021 [5,19].

Early diagnosis of exacerbation, the underlying cause, and administration of appropriate treatment are extremely important for the patient’s evolution. For the most accurate diagnosis, but also to establish the determining cause of the exacerbation, it is necessary to analyze clinical and paraclinical data. In recent years, there has been an increasing desire for treatments for various pathologies to be used depending on the phenotype of the disease. “Phenotype” defines the observable characteristics resulting from genes, environment, or the interactions between them and according to which individuals can be grouped. The importance of phenotyping derives from the need to define clusters of patients with common characteristics, characteristics that will guide specific treatment strategies in order to achieve better clinical results—“right drug, right patient, right time” [22,23,24].

Researchers in the field are trying to phenotype COPD exacerbations based on the predominance of certain characteristics such as: type of lung lesions—bronchitis or emphysema; symptomatology; type of inflammation—neutrophilic, eosinophilic, mixed; associated comorbidities, and so on. Next, we will discuss the phenotypes of COPD exacerbation in an attempt to outline the main diagnostic characteristics and therapeutic recommendations in accordance with studies in the specialized literature.

## 3. Clinical Manifestation, Diagnostic and Prognostic Biomarkers

### 3.1. Clinical Manifestation

COPD exacerbations are heterogeneous in terms of the symptoms experienced by patients, but also in terms of exacerbating factors. As specified in the AECOPD definition, exacerbations can be triggered by infectious agents as well as non-infectious factors such as pollution, allergens, climate change, and diet; also, in approximately one-third of exacerbations, the cause cannot be identified. In certain situations, clinical manifestations help clinicians establish the etiology of exacerbations [5,11,25]. The most common symptoms encountered in AECOPD are worsening dyspnea, increased sputum volume, and sputum purulence, associated with worsening cough and wheezing [5,25]. The ROMA proposal uses the VAS scale to quantify the severity of dyspnea, but it should be specified that this symptom can also be assessed by other scales such as mMRC or BORG, as will be found in the studies mentioned in this review; moreover, the impact and severity of the symptomatology can be assessed by composite scores such as the CAT scale (COPD Assessment Test) or the BODE index (Body-mass index, Obstruction, Dyspnea and Exercise) [26,27,28].

In certain situations, clinical manifestations help clinicians establish the etiology of exacerbations. Such concerns, regarding the classification and management of exacerbations according to patient symptoms, have been of interest since the last century. Depending on the symptoms presented by the patients, as well as on their mode of onset, as we will further present the results of specialized studies on this issue, the etiology of COPD exacerbations can be suspected, and the most appropriate treatment will be administered without delay.

The classification of AECOPD based on symptoms was carried out by Anthonisen in 1987. They grouped patients with AECOPD according to the symptoms presented into three subgroups as follows:

- Type 1 exacerbations—patients experience increased dyspnea, sputum volume, and sputum purulence;
- Type 2 exacerbations—patients invoke any two of the previously specified symptoms;
- Type 3 exacerbations—involves one of those symptoms in association with at least one of the following findings: worsening cough, worsening wheeze, fever without other cause, symptoms of an upper respiratory tract infection (sore throat, nasal discharge) within the past 5 days, or increase in respiratory rate or heart rate by 20% as compared with baseline [15].

By grouping patients with AECOPD in this way, the researchers observed that those with type 1 exacerbations had higher C-reactive protein (CRP) levels and a good response to antibiotic treatment compared to the other two groups [15]. Another study evaluated AECOPD according to sputum purulence, concluding that sputum purulence was associated with a sensitivity of over 90% and a specificity of over 70% in identifying a high bacterial load compared to patients with mucoid sputum. They also showed a significant increase in CRP levels in patients with purulent sputum during exacerbations, also considering sputum color as an indicator for antibiotic treatment. In patients with mucoid sputum, antibiotics were not administered during exacerbations. Thus, we can conclude that patients with purulent sputum during AECOPD are likely to benefit from antibiotic treatment [29].

Another way of phenotyping AECOPD in relation to symptoms was according to their mode of onset—sudden or gradual. Aaron et al. conducted a study conducted over a period of almost 3 years in which they used daily symptom diaries of COPD patients to characterize the temporal evolution of the onset of exacerbations. Thus, they divided exacerbations into two distinct patterns, with sudden onset and with progressive onset, and statistical analysis revealed that patients with sudden-onset COPD presented greater mean daily symptom scores, greater peak symptom, earlier peak symptoms, and shorter median recovery times back to baseline health status compared to patients with gradual onset of exacerbation. The authors assumed that the triggers of sudden-onset AECOPD were infectious in nature—viral or bacterial—based on the symptoms identified. However, exacerbations with viral symptoms during the winter months had a gradual onset with prolonged recovery. Regarding the treatment used during exacerbations, it did not differ between the two groups, being represented by antibiotics and steroids or a combination of the two, but those with sudden-onset exacerbations tended to receive less treatment compared to the other group [30].

Even though the studies described above are older, their results remain consistent with recent studies. Therefore, the main symptoms experienced during exacerbations are those described by Anthonisen. Worsening dyspnea is most commonly seen in these patients, along with increased sputum production. Also, the intensity of dyspnea is greater in patients with frequent exacerbations, and the symptomatology is more extensive in patients with moderate and severe exacerbations. The presence of purulent sputum seems to be associated with exacerbations due to bacterial infections [5,31,32,33,34,35,36].

With an awareness of the prodromal phase of an exacerbation, which often correlates with the triggering factor of the exacerbation, but also of the time required for the exacerbation to resolve, we can act as quickly as possible in administering appropriate treatment. It is obvious that by correlating symptoms with bacteriological investigations in sputum and biological ones when possible, the diagnosis of AECOPD is made more accurate.

Given the non-specific nature of these symptoms, when we are faced with a patient suspected of having AECOPD, it is important to evaluate the decompensation of other associated pathologies (pneumonia, asthma, interstitial lung diseases, high blood pressure, arrhythmias, congestive heart failure, pulmonary embolism, and so on) that may mimic AECOPD [5,11].

### 3.2. Diagnostic and Prognostic Biomarkers

The identification of a specific biomarker for the diagnosis of AECOPD remains of interest to researchers because no such marker has been identified to date. This is most likely due to the heterogeneity of these acute events that occur in the evolution of this respiratory disease. Moreover, such a biomarker would also be expected to predict prognosis, as well as the possibility of guiding treatment and evaluating response to treatment. During AECOPD, the level of systemic and local inflammation in the airways increases, being very heterogeneous [13,37,38]. Analyzing studies in the specialized literature, over time, several biological markers have been analyzed with an aim to identify the etiology and to phenotype AECOPD so as to choose the best therapeutic regimen in relation to the etiological factor. The most studied biomarkers for assessing systemic inflammation in AECOPD are C-reactive protein (CRP) [39,40,41], procalcitonin (PCT) [42,43], serum amyloid A [44], fibrinogen [45], inflammation cell chemotactic factor [36], and cytokines (IL-6, TNF) [46].

Exacerbations with infectious etiology are the most common among COPD patients, representing between 70–80% of them. Papi A et al. identified, in their study, the presence of bacteria in 30% of patients with AECOPD, viruses in 23%, and bacterial–viral co-infection in 25% of patients. The most common bacteria identified by quantitative cultures are *Haemophilus influenzae*, *Streptococcus pneumoniae*, *Moraxella catarrhalis*, *Staphylococcus aureus*, and *Pseudomonas aeruginosa*. The most common viruses identified by PCR (polymerase chain reaction) are represented by rhinoviruses, influenza viruses, respiratory syncytial viruses, parainfluenza viruses, and coronaviruses [36,45,47,48]. As can be seen below, there are markers that tend to have higher values during bacterial infections and markers that are more specific to viral infections.

C-reactive protein, a non-specific marker of infection, inflammation, and injury, is one of the most studied markers in AECOPD. Numerous studies have shown a correlation between CRP levels and AECOPD, some of them demonstrating that elevated values of this marker are associated with bacterial exacerbations. Peng C et al. showed in their study that a CRP value above 19.65 mg/L has a sensitivity of 78% and a specificity of 84% in establishing the bacterial infectious cause of AECOPD. Moreover, they found that a CRP value above 15.2 mg/L in patients with mucoid sputum is indicative of a bacterial infectious exacerbation, with a specificity and sensitivity of approximately 80% [49].

Another study showed similar results to Peng’s, namely that CRP levels were significantly higher in patients with positive cultures for bacteria (58.30 mg/L) compared to those in whom viruses (37.3 mg/L) were identified as exacerbating agents. The highest CRP values (74.5 mg/L) were in patients in whom *H. influenzae* and *Streptococcus pneumoniae* were identified. Furthermore, they showed that CRP values above 100 mg/L were associated with a fourfold increased risk of hospitalization [50]. Another real-world study confirmed the correlation between elevated CRP values and positive cultures for potentially pathogenic microorganisms [51]. Noell et al. demonstrated in their study that the distinction between acute exacerbation and the period of stability in COPD patients could be achieved by identifying increased values of CRP, leukocytes and worsening dyspnea [52].

Procalcitonin is another biomarker that can differentiate between bacterial and viral exacerbations. Nseir S et al. showed that a PCT value above 0.5 mg in patients with severe AECOPD is independently associated with the isolation of bacteria in cultures [53]. In patients with severe COPD requiring intubation and invasive ventilation, PCT levels are independently associated with an increased risk of intensive care unit mortality [43]. PCT levels can guide antibiotic treatment in patients with AECOPD, thus reducing its inappropriate use [54,55], but there are cases in which PCT is negative in patients with bacterial infectious exacerbations that require antibiotic therapy [56]. For these reasons, other inflammatory markers such as CRP should be analyzed and the need for antibiotic treatment in patients with signs of bacterial infection and negative PCT should be carefully weighed.

Bafadhel M et al. measured multiple inflammatory markers in serum in their study, obtaining increased values during AECOPD of IL-6, tumor necrosis factor (TNF) receptors I and II, serumamyloid-A, CRP, procalcitonin, C-X-C motif chemokine 10 (CXCL10) and serum eosinophil cationic protein [24]. These biomarkers can show the severity of exacerbation and may have prognostic value, but no cut-off values are defined that allow clear differentiation between various phenotypes of AECOPD [25]. Viral AECOPD is associated with elevated serum levels of CXCL10 (a serum CXCL10 cut off of 56 pg/ml have a sensitivity of 75% and specificity of 65%) [24], fibrinogen and IL-6 [45,48].

The level of airway inflammation increases during AECOPD, and the type of inflammation can be assessed by fractional exhaled nitric oxide (FeNO) and induced sputum analysis [25]. Based on the type of inflammatory cells identified in the sputum, Gao P et al. grouped patients with AECOPD into four subgroups—eosinophilic (sputum eosinophils are >2.5% of total cells), neutrophilic (neutrophils > 61%), mixed granulocytic (eosinophils > 2.5% and neutrophils > 61%), and paucigranulocytic (eosinophils ≤ 2.5% and neutrophils ≤ 61%). Following statistical analysis, they found differences between phenotypes both symptomatically and biologically. Patients with AECOPD and neutrophilic or mixed inflammation had a higher BODE score, more sputum inflammatory cells, lower lung function, and longer hospital stay, accompanied by higher concentrations of sputum matrix metalloproteinase-9, IL-6, and CRP, and also higher concentrations of serum amyloid A, IL-6, and CRP. Moreover, 83% of patients with neutrophil predominance in sputum displayed evidence of bacterial infection [57]. Another study showed that neutrophils increase in sputum regardless of whether the exacerbating agent is bacterial or viral, but eosinophils increase predominantly in viral exacerbations [36]. Bafadhel et al. showed in their study that sputum IL-1b, with a cut-off value of 125 pg/mL, had a sensitivity of 90% and a specificity of 80% in identifying exacerbations caused by bacterial infections [24].

FeNO dosing can provide information about the response to corticosteroid treatment, as it correlates with eosinophilic inflammation [25]. There are studies that have shown a correlation between high FeNO levels and improvement in FEV1 after corticosteroid therapy [58,59,60]. Even though there are studies that show an increased level of FeNO during COPD exacerbations compared to the stable period of the disease, the use of this marker in clinical practice to guide treatment remains limited because in most studies, a correlation between FeNO levels and lung function impairment was not identified [61,62,63].

Recently, there has been increasing interest in functional assessment during COPD exacerbations through the forced oscillation technique. This is an effort-independent lung function test to assess respiratory impedance (resistance and reactance) and it can be used to detect expiratory limitation (EFL) in AECOPD patients [64,65]. Alqahtani J.S et al. showed in their study that patients with EFL had lower FEV1 and forced vital capacity, and they also observed that during recovery from exacerbation, EFL changed in line with the improvement in dyspnea. Therefore, they concluded that this investigation can be used routinely to evaluate and monitor patients requiring hospitalization during COPD exacerbations [66]. However, further studies are needed to validate the utility of the forced oscillation technique in these cases.

Regarding the prediction of the risk of exacerbations, there are studies that have shown that certain medical events and biomarkers are associated with an increased risk of exacerbation compared to COPD patients who did not have those characteristics. Hurst et al. showed that the number of previous exacerbations, forced expiratory volume in one second (FEV1), history of heartburn or gastroesophageal reflux, and quality of life were the most important factors for predicting the risk of future exacerbations. In this study, as well as in another extensive study that included a large number of biomarkers, it was demonstrated that the value of these markers did not add value to the prediction of exacerbations compared to clinical and functional characteristics [13,67,68]. However, Thomsen et al. showed in their study that included over 6000 patients that the simultaneous presence of elevated CRP, fibrinogen, and leukocyte values was associated with an increased risk for AECOPD, with a predilection for patients with low FEV1 and those with a history of exacerbations [69].

To simplify the data presented about the markers used in identifying the etiology of exacerbations, we can state the following:

- Increased serum levels of CRP and/or positive procalcitonin are associated with the bacterial etiology of exacerbations;
- IL-6, fibrinogen, and CXCL10 in serum are associated with the identification of viruses as determining factors of AECOPD;
- The presence of neutrophils and IL-1b in induced sputum correlates with bacterial exacerbations;
- The presence of eosinophilic inflammation in the respiratory tract is associated with the viral nature of AECOPD.

In conclusion, it can be observed that the dosage of serum and sputum markers provides useful information in the phenotyping of COPD exacerbations, thus guiding the administration of optimal treatment and being predictors of the evolution and prognosis of patients. However, further studies are needed to validate more specific biomarkers in phenotyping AECOPD, establishing the etiology of exacerbations, and managing these episodes.

## 4. COPD Exacerbation Phenotypes

### 4.1. Non-Exacerbator Phenotype vs. Emphysema or Chronic Bronchitis Frequent Exacerbator Phenotype

Non-exacerbator phenotype defines patients who have had fewer than two moderate or severe COPD exacerbations in the past 12 months [22,70,71]. This phenotype is more common, accounting for approximately 60% of COPD patients according to studies in the literature [72,73]. Patients with non-exacerbating COPD phenotype have fewer symptoms, a lower risk of lung function deterioration and impaired quality of life, and a lower risk of mortality compared to patients with the exacerbating phenotype [70,72,73]. The treatment of these patients aims to maintain clinical stability, with the administration of one or two bronchodilators being recommended. Non-pharmacological measures should also be associated, these being represented by quitting smoking, inclusion in respiratory rehabilitation programs, and self-management [22,71].

The frequent exacerbator phenotype is defined by the presence of two or more moderate exacerbations (requiring at least outpatient treatment with systemic corticosteroids and/or antibiotics) in the past year or at least one severe exacerbation requiring hospitalization [22,74,75]. Patients with frequent exacerbations of COPD tend to be men, and also to be older, present higher levels of both systemic and pulmonary inflammation, be associated with lower forced expiratory volume in 1 s percent predicted (FEV1) and rapid functional decline, have more intense symptoms that cause a decrease in quality of life, have a history of previous exacerbations, and have higher mortality compared to patients with infrequent exacerbations [25,76,77,78,79,80,81,82]. The frequent exacerbating phenotype was divided into the emphysematous exacerbating phenotype and the chronic bronchitis exacerbating phenotype. The chronic bronchitis phenotype is defined by the presence of cough and sputum production for at least 3 months during two consecutive years, and the emphysematous phenotype defines patients without chronic bronchitis and with radiological (computed tomography scan or chest X-ray) and/or functional (reduced CO diffusion) evidence of emphysema [22,70,83,84]. Given that the management of these phenotypes is different, it is important to classify patients into the correct phenotype in order to administer the correct treatment [22,83].

- Emphysema frequent exacerbator phenotype

This phenotype has a prevalence of approximately 10%, but patients with this form of the disease have the highest mortality rate compared with other COPD phenotypes. Exacerbations of this phenotype are due to hyperinflation, not inflammation and respiratory infection, which occurs in patients with chronic bronchitis [22]. For these reasons, the detection of emphysema through imaging or respiratory function methods should be facilitated; moreover, the suspicion of the emphysematous phenotype can be made clinically, these patients being dyspneic, not presenting cyanosis, and having a low body mass index [70,71,72,73].

In the study conducted by Berat Uslu, patients with frequent exacerbations had more frequent emphysema on chest CT compared to patients in the non-exacerbation group. Furthermore, these patients presented with a low number of eosinophils in the blood and increased pulmonary functional impairment (low FEV1, FVC, and FEV1/FVC) [82]. Similar results regarding the relationship between the presence and severity of emphysema in COPD patients and frequent exacerbations were also identified in the study by Dan Zhu et al. [79].

The recommended treatment for patients with emphysema and frequent exacerbations is with long-acting bronchodilators (mono or dual bronchodilator depending on the severity of ventilatory dysfunction). Inhaled corticosteroids (ICS) are to be avoided in these cases because airway inflammation is reduced and further increases the risk of pneumonia. ICS can be associated in patients with a history of asthma, atopy, and/or blood eosinophilia [12,22,25,83].

- Chronic bronchitis frequent exacerbator phenotype

The prevalence of the chronic bronchitis phenotype is approximately 20%. Patients with chronic bronchitis have a significantly higher risk for moderate or severe COPD compared to COPD patients without chronic bronchitis. In the case of this patient phenotype, it is necessary to exclude bronchiectasis by performing high-resolution CT for correct classification of the phenotype. It is also recommended to perform a bacteriological examination of sputum to identify chronic microbial infections [22,72,73].

An extensive meta-analysis showed that patients with an exacerbating phenotype associated with chronic bronchitis have lower lung function and more intense symptoms as quantified by increased modified Medical British Research Council (mMRC) and COPD assessment test (CAT) scores compared to patients with a non-exacerbating COPD phenotype [80]. These patients also associate the presence of more comorbidities compared to other phenotypes, especially depression and anxiety [85]. The chronic bronchitis frequent exacerbator phenotype is the second most common after the non-exacerbating COPD phenotype, occurring in approximately 20–30% of COPD patients according to studies [72,73,86].

Patients with COPD with a bronchitic phenotype should be treated with long-acting bronchodilators, which can be combined with ICS [22,25,83]; they also respond well to phosphodiesterase-4 (PDE-4) inhibitors (roflumilast) combined with inhaled therapy compared to patients with an exacerbating emphysematous phenotype [85,87,88,89]. In the case of those who are adequately treated with inhaled medications but experience multiple exacerbations within a year (at least three exacerbations) and have severe airway obstruction, long-term treatment with macrolide antibiotics may be associated with the aim of reducing the number of exacerbations [90,91,92]. This phenotype of patients can also be administered mucolytics (acetylcysteine, erdosteine), with especially favorable results recorded in patients with at least two exacerbations per year and severe ventilatory dysfunction (FEV1 < 50%), especially if they have a contraindication for ICS [12,93,94,95].

### 4.2. Comorbidity Phenotypes

COPD, but especially AECOPD, is associated with comorbidities. Considering that COPD is a pathology that evolves with both local and systemic inflammation, the comorbidities are both pulmonary and extrapulmonary [25]. The most common pulmonary comorbidities associated with COPD are asthma, pneumonia, bronchiectasis, obstructive sleep apnea, and lung cancer [96,97,98]. Regarding extrapulmonary comorbidities, these are multiple, the most important being cardiovascular disorders, skeletal muscle dysfunction, osteoporosis, diabetes, depression, and anxiety [99,100,101,102,103].

Patients with COPD and associated comorbidities have a significantly higher risk of exacerbations compared to patients with COPD without comorbidities. In terms of AECOPD, the presence of comorbidities prolongs the length of hospital stay, increases mortality, and is associated with unfavorable outcomes in both the short and long term [25,74].

Next, we will provide some data about some of the many comorbidities that can be associated with COPD.

Asthma—COPD phenotype defines patients with COPD who also present diagnostic criteria for asthma or who present features considered as suggestive of asthma, such as personal history of asthma, a strongly positive BD test (increase in FEV1 > 400 mL and 15%), a history of atopy, fractional exhaled nitric oxide > 40 ppb, and/or peripheral blood eosinophilia > 300 cells per mm^3^ [22,70,104,105]. During AECOPD, patients with both diseases present symptoms such as cough with mucous but not purulent sputum, wheezing, and rhinitis phenomena. The presence of eosinophils is associated with Th2-type inflammation, inflammation predominantly found in asthma and with a good response to corticosteroids. For these reasons, the treatment of these patients involves the association of ICS with bronchodilators. The clinical and functional response of these patients to ICS is good because they present a high level of eosinophilic inflammation in the airways [84,106,107]. In the case of those in whom frequent exacerbations persist despite maximal inhaler treatment, the introduction of biological therapy with anti-IgE, anti-IL 5 antibodies, anti-IL 5 receptor, and anti-IL 4/IL 13 receptor may be considered depending on the positivity of the markers used in asthma phenotyping [84,108,109]. In a phase 3 study, patients with COPD and type 2 inflammation who received treatment with dupilumab experienced fewer exacerbations, better lung function and quality of life, and less severe respiratory symptoms than those in the placebo group [110]. Benralizumab also showed a decrease in the number of exacerbations in patients with COPD and characteristics of type 2 inflammation [111].

The association of COPD with bronchiectasis defines a distinct phenotype, being more common in male patients and heavy smokers. These patients have a higher risk of frequent exacerbations, presenting increased daily sputum, more impaired lung function, increased level of inflammation, chronic colonization with potentially pathogenic microorganisms, and increased risk of chronic colonization with various microorganisms such as *Pseudomonas aeruginosa*, but also higher mortality [5,112,113]. Regarding treatment, associated with bronchodilators, the administration of mucolytics is recommended; also, in chronically colonized patients or those with repeated respiratory infections, broad-spectrum antibiotic therapy may be necessary for longer periods of time. The administration of ICS must be carefully balanced since in patients with repeated respiratory infections or bacterial colonization, it could maintain the infectious processes without bringing any benefit [5,25,114,115]. These patients should also benefit from bronchial drainage through mechanical mucus-clearing methods such as positive expiratory pressure, flutter valves, or high-frequency chest compression vests or chest physiotherapy. Further studies on larger populations are needed to validate the benefits of these techniques. Their application should be restricted to well-selected patients [22,116,117].

The presence of extrapulmonary comorbidities in patients with COPD is a risk factor for exacerbation of respiratory disease; also, frequent exacerbations of COPD increase the risk of decompensation of associated pathologies, thus increasing the risk of death in these patients.

A study conducted by Arostegui I. in 16 hospitals in Spain classified patients with AECOPD into four subtypes based on clinical severity and the presence or absence of comorbidities. Through this classification, they aimed to understand the pathophysiological mechanisms involved in exacerbations to establish more effective therapeutic strategies and improve the patient care process. The four AECOPD patient subtypes were defined from A to D and presented the following characteristics: subtype A (n = 934), neither high comorbidity nor severe exacerbation; subtype B (n = 682), moderate comorbidities; subtype C (n = 562), severe comorbidities related to mortality; and subtype D (n = 309), very severe process of exacerbation, significantly related to mortality and admission to an intensive care unit. The hospitalization rate was over 50% in all groups, being significantly higher in groups C and D. Also, patients in group D had the highest rate of intensive care admission, the highest need for non-invasive mechanical ventilation, and the highest mortality, followed by group C in terms of these needs. These results could guide the classification of patients with AECOPD into a specific risk phenotype, guiding therapeutic interventions [118].

## 5. Conclusions

This narrative review provides an overview of the most commonly encountered phenotypes of COPD exacerbation in clinical practice, as well as the recommended therapeutic measures depending on the phenotype. Considering that AECOPD is an extremely heterogeneous disease, the application of the phenotyping concept allows for an individualized approach to patients regarding therapeutic measures and their management with the aim of reducing the number of acute events, as well as avoiding the adverse effects of treatments used in excess without bringing benefit to the patient. Despite the multitude of studies conducted to date, additional studies are needed that include larger patient populations to validate the results referring to certain diagnostic markers, as well as various therapeutic modalities. Considering that this concept is increasingly used in the medical world, we believe that it should be used by all clinicians so that patients with AECOPD benefit from appropriate treatment.

**Figure 2:**
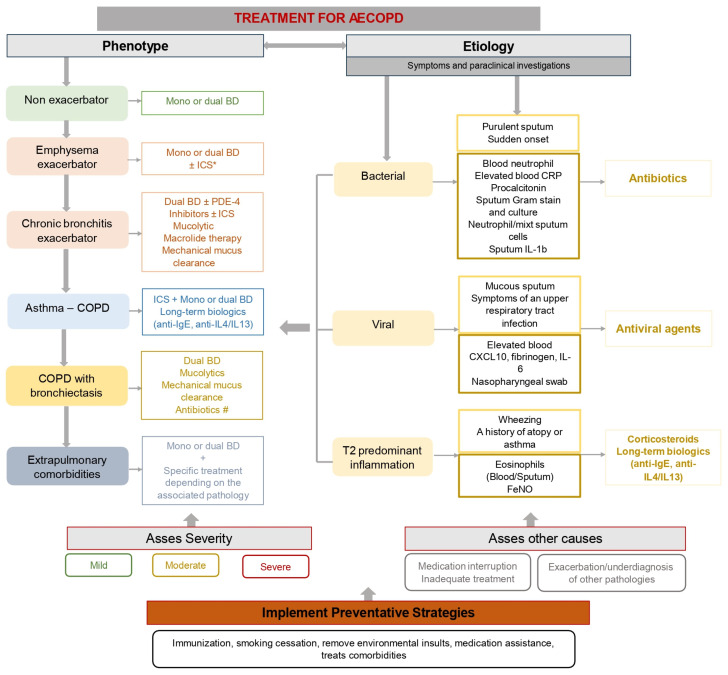
Management of COPD exacerbations. * ICS can be associated in patients with a history of asthma, atopy, and/or blood eosinophilia. # Antibiotic therapy may be required for longer periods of time in patients with chronic colonization or recurrent respiratory infections. Abbreviations: BD—bronchodilator; CRP—C-reactive protein; AECOPD—exacerbation of chronic obstructive pulmonary disease; FeNO—fractional exhaled nitric oxide; ICS—inhaled corticosteroid; PDE—phosphodiesterase.

In Figure 2, we have exemplified the therapeutic measures used in the management of COPD exacerbations depending on the phenotype of the disease but also depending on the etiological factor of the exacerbation; also as can be seen from this figure, the management of COPD involves different measures depending on the severity of the acute episode, and the assessment of associated comorbid situations is mandatory. Moreover, measures to prevent exacerbations must be implemented regardless of the phenotype and etiology of COPD.

## Abbreviations

The following abbreviations are used in this manuscript:

AECOPD	Acute exacerbations of chronic obstructive pulmonary disease
BODE	Body-mass index, Obstruction, Dyspnea and Exercise
CAT	COPD Assessment Test
COPD	Chronic obstructive pulmonary disease
GOLD	Global initiative for chronic obstructive lung disease
VAS	Visual analog dyspnea scale
RR	Respiratory rate
HR	Heart rate
SaO_2_	Oxygen saturation
ABG	Arterial blood gases
PaO_2_	Arterial pressure of oxygen
SABD	Short acting bronchodilators
CS	Corticosteroids
CRP	C-reactive protein
PCT	Procalcitonin
CXCL10	C-X-C motif chemokine 10
FeNO	Fractional exhaled nitric oxide
ACO	Asthma—COPD phenotype
BD	Bronchodilator
ICS	Inhaled corticosteroid
PDE	Phosphodiesterase
